# The Color Nutrition Information Paradox: Effects of Suggested Sugar Content on Food Cue Reactivity in Healthy Young Women

**DOI:** 10.3390/nu12020312

**Published:** 2020-01-24

**Authors:** Jonas Potthoff, Annalisa La Face, Anne Schienle

**Affiliations:** Institute of Psychology, University of Graz, Universitaetsplatz 2, 8010 Graz, Austria; annalisa.la-face@uni-graz.at (A.L.F.); anne.schienle@uni-graz.at (A.S.)

**Keywords:** nutrition facts, food cue reactivity, sugar, eye tracking, priming, color

## Abstract

Color nutrition information (CNI) based on a traffic light system conveys information about food quality with a glance. The color red typically indicates detrimental food characteristics (e.g., very high sugar content) and aims at inhibiting food shopping and consumption. Red may, however, also elicit cross-modal associations with sweet taste, which is a preferable food characteristic. We conducted two experiments. An eye-tracking study investigated whether CNI has an effect on cue reactivity (dwell time, saccadic latency, wanting/liking) for sweet foods. The participants were presented with images depicting sweets (e.g., cake). Each image was preceded by a colored circle that informed about the sugar content of the food (red = high, green = low, gray = unknown). It was tested whether the red circle would help the participants to direct their gaze away from the ‘high sugar’ item. A second experiment investigated whether colored prime circles (red, green, gray) without nutrition information would influence the assumed sweetness of a food. In Experiment 1, CNI had the opposite of the intended effect. Dwell time and saccadic latency were higher for food items preceded by a red compared to a green circle. This unintended response was positively associated with participants’ liking of sweet foods. CNI did not change the wanting/liking of the displayed foods. In Experiment 2, we found no evidence for color priming on the assumed sweetness of food. Our results question whether CNI is helpful to influence initial cue reactivity toward sweet foods.

## 1. Introduction

Food is a primary reinforcer that automatically captures visual attention. This evolutionary-based mechanism assists with the localization of food sources within the environment and, in turn, enables sufficient caloric uptake by the individual [1]. Studies utilizing neurophysiological measures and eye-tracking have shown that the human attention system very quickly identifies visual food cues and differentiates them from non-food objects [2,3,4]. Additionally, high-calorie food captures more attention than low-calorie food [5,6].

The increased attention to cues of high-calorie food has become problematic in Western countries because the exposure to such stimuli triggers the urge to eat [7]. Food cues and (high-calorie) foods are almost omnipresent in our everyday lives. Therefore, a link between individual food cue reactivity (FCR), overeating, and weight gain is not surprising [7].

In order to reduce the shopping and consumption of high-calorie food, effective interventions that are able to reduce FCR are urgently needed. It has already been demonstrated that nutritional knowledge is able to influence FCR [8]. A number of studies has found a positive correlation between nutritional knowledge and healthy dietary habits [9,10,11,12,13,14]. The knowledge transfer about the sugar content of food seems to be a promising starting point for such interventions because large proportions of calories are consumed in the form of sugar [15]. Moreover, the excessive consumption of sugary food is associated with an increased risk of cardiovascular disease, cancer, and diabetes [16]. However, findings regarding the relationship between individual knowledge about the sugar content of specific foods and actual consumption are heterogeneous [17,18,19]. Therefore, it seems likely that knowledge about the sugar content of food cannot always be accessed easily and quickly enough [20,21,22,23].

Therefore, color nutrition information (CNI) based on a traffic light system seems to be an efficient method to convey information about food quality. This system is already used in front of pack food labels [24]. The color red (as a stop signal) typically indicates detrimental food characteristics (e.g., very high sugar content), whereas green signals positive features [25,26,27,28].

However, even though the traffic light system is widely used, little is known about how CNI influences initial food cue reactivity. Furthermore, little is known about possible unintended effects of the commonly used colors (red, green). The color red may elicit cross-modal associations with sweet taste, which is a preferable food characteristic [29,30]. For example, cider was perceived as sweeter when served in a bottle with a red label compared to a green label [31]. The red-sweetness association seems to be stronger for drinks compared to solid foods. Lemos et al. [32] presented colored prime stimuli (red, green, amber cycles) that were followed by an image of a salty or sweet food item. The seven sweet food items used in this experiment were, on average, rated as more positive (hedonic valence) after the presentation of a red cycle compared to a green cycle. This effect was most pronounced for the only liquid (a carbonated soft drink) used as stimulus material. However, for half of the solid sweet foods, the hedonic valence was actually lower after the presentation of a red cycle compared to a green one. Based on this previous research, it remains unclear whether red color used in food labels as ‘warning signals’ implicitly primes sweet taste associations.

The aim of the present investigation was twofold. We investigated effects of colored nutrition information (traffic light symbols indicating the sugar content) on initial food cue reactivity (Experiment 1). In a second experiment, we investigated priming effects of the colors red and green on assumed sugar content/sweet taste (Experiment 2).

## 2. Materials and Methods

### 2.1. Sample

Experiments 1 and 2 were conducted following the rules of the Declaration of Helsinki of 1975, revised in 2013. The experiments were approved by the ethics committee of the University of Graz (ethical approval code: 39/31/63 ex 2018/19).

#### 2.1.1. Sample Experiment 1

Fifty-one women (mean age: 22.0 years, SD = 2.99; range 18–33) with a body mass index (BMI) of *M* = 22.5 (SD = 3.85) took part in this study. We selected women because previous research has suggested that the use and understanding of nutrition information is related to demographic characteristics, notably social grade, age, and gender [33]. Participants had normal or corrected-to-normal vision and did not report any current medication or mental disorder. Forty-nine participants were university students, and the other were white-collar workers. Participants were recruited via email lists and postings at the university campus as well as dormitories. Psychology students (*N* = 32) received course credits for their participation. Sample characteristics are displayed in Table 1.

#### 2.1.2. Sample Experiment 2

A total of 99 participants (age: *M* = 25.03 years, SD = 6.17 years; BMI: *M* = 22.61 kg/m^2^, SD = 2.81 kg/m^2^) completed an online experiment. Of the participants, 55 had a high school diploma, 44 participants graduated from college. The majority of participants was female (female: *N* = 74, male: *N* = 25).

### 2.2. Stimuli and Design Experiment 1

We presented color nutrition information (CNI) that reflected the sugar content of a specific food item (green: Low sugar content, red: High sugar content, gray: Unknown sugar content; diameter: 354 pixels) and 48 pictures of sweet foods (e.g., cakes, ice cream, candies from the FoodPics database [34]). Each picture had a size of 600 × 450 pixels. Food images and CNI were presented on a white background on an LCD screen. We selected food products of which low sugar versions are commonly available on the market. We assigned 16 images to each category (low/high/unknown sugar content) and created three parallel versions of the experiment. Due to the parallel versions, each image was suggested to have a low, high, or unknown sugar-content for one-third of the participants. Participants were randomly assigned to one of the three parallel versions.

**Figure 1:**
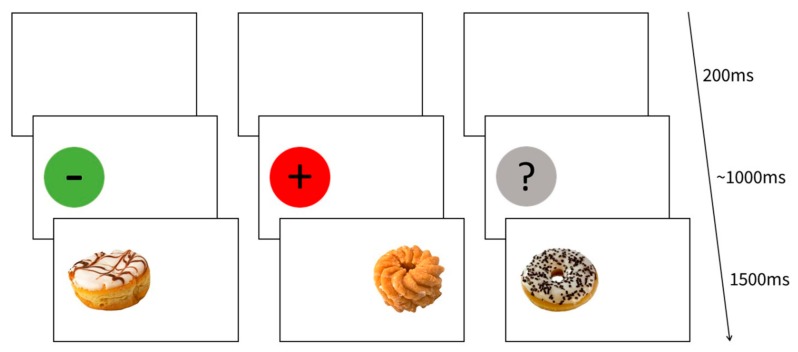
Example trials for (from left to right) low sugar label, high sugar label, and unknown sugar label. Each food image was presented twice: Once in the same location as the label (example: low sugar & unknown sugar) and once in the peripheral location (example: high sugar label). In 50% of trials, the label was presented on the left side of the screen. In the other 50%, the label was presented on the right side of the screen (not displayed here).

At the beginning of each trial, a circle was presented on either the center of the left or the right half of the screen. As soon as participants were gazing at it steadily for 1000 ms, the CNI disappeared and the allocated food image was presented for 1500 ms. Each pair of CNI and food image was shown in two trials: Once the food image appeared in the same location as the CNI (current gaze location: Figure 1), the other time the food image was presented on the opposite side of the screen (peripheral location), resulting in 96 trials (16 per suggested sugar content: Low, high, unknown; and position: Current gaze location, peripheral). Trials were followed by an intertrial interval of 200 ms. The trial order was randomized.

The participants were instructed to inspect the circles. Throughout the paradigm, two food items of each category were presented in the center of the screen. Participants were asked to rate these food items regarding their specific appetite (“How much would you like to taste this food right now?” 0: “Not at all”, 6: “Very much”) and general liking (“How much do you like this food in general?” 0: “Not at all”, 6: “Very much”).

### 2.3. Procedure Experiment 1

After providing written informed consent, participants read a short info sheet about color-coded nutrition facts (high sugar/red symbol: Above 12.5 g sugar per 100 g food, low sugar/green symbol: Below 5 g sugar per 100 g food). Subsequently, participants rated their general appetite and hunger on a seven-point scale (appetite: 0: “I have no appetite at all.”, 6: “I have an extreme urge to eat something right now.”; hunger: 0: “I have no hunger at all.”, 6: “I am extremely hungry.”). Furthermore, participants rated their preference for sweet food (“How much do you like sweet food in general?”, 0: “Not at all.”, 4: “Very much.”). Subsequently, the eye-tracking paradigm described above was conducted.

Following the eye-tracking paradigm, participants conducted a survey about their demographics and the following questionnaires.

### 2.4. Questionnaires Experiment 1

The participants completed the Eating Disorder Examination-Questionnaire (EDE-Q; [35]) and the Impulsivity Short Scale (I-8; [36]). The EDE-Q consists of 41 items (e.g., “Were you afraid to lose control over your eating?”) that are answered on seven-point scales (0: “Not at all”, 6: “Very much”) and are concerned with the previous four weeks. Furthermore, the EDE-Q inquires weight and size (i.e., BMI). In the present sample, Cronbach’s alpha for the EDE-Q was α = 92. The I-8 consists of eight items (e.g., “I usually think carefully before I act.”), which are answered on five-point scales (1: “Doesn’t apply at all”, 5: “Applies completely”; Cronbach’s α = 75 for the I-8). 

The questionnaires were selected because disordered eating and impulsivity have been associated with elevated food cue reactivity in previous research [37].

### 2.5. Eye Movement Recording and Analysis Experiment 1

Two-dimensional eye movements were recorded using an SMI RED250mobile eye-tracker with a sampling rate of 250 Hz. Head movements were minimized by a chin rest. We calibrated both eyes and analyzed data from the eye that produced the better spatial resolution (typically more accurate than a 0.35° visual angle). Stimuli were presented on a white background on a 24-inch screen with a resolution of 1920 × 1080 pixels. The viewing distance was 60 cm, resulting in a size of 15.6° × 11.7° viewing angle for food images and a diameter of 9.2° viewing angle for CNIs. The experiment was controlled using the SMI Experiment Center (Version 3.6.53, SensoMotoric Instruments, Teltow, Germany). For event detection, standard thresholds of the SMI BeGaze Software (Version 3.6.52, SensoMotoric Instruments, Teltow, Germany) for high speed eye-tracking data (recommended for sampling rate > 200 Hz) were used to detect saccades (velocity threshold: 40°/s). Data were exported using SMI BeGaze and customized Python scripts. Within BeGaze, we defined the food images as areas of interest (AOI). We conducted gaze data analysis exclusively for the food AOI of each trial. We defined the dependent variable, ‘saccadic latency’, as the time from stimulus onset to the start of the first saccade that ended outside of the food AOI. Saccadic latency was calculated only for trials with the participants’ gaze position within the AOI at stimulus onset (CNI was presented in the same position as the subsequently presented food). Saccadic latency therefore measured how long it took participants to actively relocate their gaze away from a food item.

The second dependent variable, ‘dwell time’, was defined as the sum of fixation durations within the AOI. Other than saccadic latency, we computed dwell time for all trials (trials in which the food appeared at gaze location, as well as trials in which food appeared in the peripheral location).

### 2.6. Stimuli and Design Experiment 2

Thirty pictures of sweet food from Experiment 1 (size: 600 × 450 pixels) were presented in the center of the computer screen for 1500 ms each. Prior to the picture presentation, one of three colored circles (red, green, gray) was shown. The circles (diameter: 354 pixels) were displayed centrally on a white background for 1000 ms. The circles did not contain any text and were presented without any further instructions. We created three subsets of prime-stimulus combinations to ensure that each picture was preceded by a red, green, or gray circle. The participants were randomly assigned to one of the three color-food combinations (combination 1: *N* = 28, combination 2: *N* = 38, combination 3: *N* = 33). There was no significant difference between groups in mean age (*F*(2,96) = 0.17, *p* = 0.84, *η*2*p* = 0.004), BMI (*F*(2,67) = 1.64, *p* = 0.20, *η*2*p* = 0.047), hunger level (*F*(2,96) = 0.83, *p* = 0.44, *η*2*p* = 0.02), or gender distribution (Chi^2^ (2, *N* = 99) = 1.52, *p* = 0.47).

After the presentation of each food image, the participants rated the assumed sweetness of the food on a scale from 0% (“not sweet at all”) to 100% (“extremely sweet”). Additionally, the valence of two food images per color was rated (0%: “Extremely unpleasant”, 100%: “Extremely pleasant”). The trials were presented in random order.

### 2.7. Procedure Experiment 2

Participants were asked to conduct the experiment at home without distraction on a computer with a (hardware) keyboard and mouse. After giving informed consent, participants provided demographic data (age, education, gender). They reported their current hunger level (“How hungry are you right now?” 0: “Not hungry at all”, 6: “Extremely hungry”), weight, and height. Subsequently, the participants were presented with 30 images of sweet food in randomized order. The experiment was conducted using Pavlovia and was programmed in Python using PsychoPy 3.2.2 [38].

### 2.8. Statistical Analysis

Repeated measures analyses of variance (ANOVAs) were computed to test the effect of CNI (low, high, unknown sugar content) on specific appetite, general liking of the displayed food items, and dwell time spent on food images, as well as saccadic latency away from food. For trials in which the food image was presented in the periphery of the current gaze, the repeated measures ANOVA was conducted only for dwell time (Experiment 1). In Experiment 2, ANOVAs were conducted to test the effect of color. If sphericity was violated (Mauchly’s Test of Sphericity), Greenhouse–Geisser correction was applied. We reported the effect size as *η*2*p* (partial eta squared) and Holm adjusted p-values. The *p*-values smaller than 0.05 were considered statistically significant. Data are available online at OSF (OSF Project DOI: 10.17605/OSF.IO/FJ3UZ, Center for Open Science, Charlottesville, VA): www.osf.io/g4d7s/

## 3. Results

### 3.1. Results Experiment 1

#### 3.1.1. Questionnaire Data

Participants obtained an average EDE-Q score of *M* = 1.33 (SD = 0.96), which did not differ significantly from the mean (*M* = 1.44) of the healthy norm sample (individuals without any current diagnosis of an eating disorder, *N* = 409, [35]), *t*(50) = 0.80, *p* = 0.43, *d* = 0.11. The mean I-8 score of the present sample of *M* = 2.63 (SD = 0.62) did not differ significantly from the average impulsivity of the female norm sample aged between 18 and 35 years (*M* = 2.62), *t*(50) = 0.09, *p* = 0.93, *d* = 0.01.

#### 3.1.2. Appetite and General Liking of Presented Food Images

CNI had no statistically significant effect on reported appetite (*F*(2,100) = 0.38, *p* = 0.68, *η*2*p* = 0.008) and general liking of the depicted food items (*F*(2,100) = 0.58, *p* = 0.56, *η*2*p* = 0.01; see Table 1).

#### 3.1.3. Eye Movements

**Figure 2:**
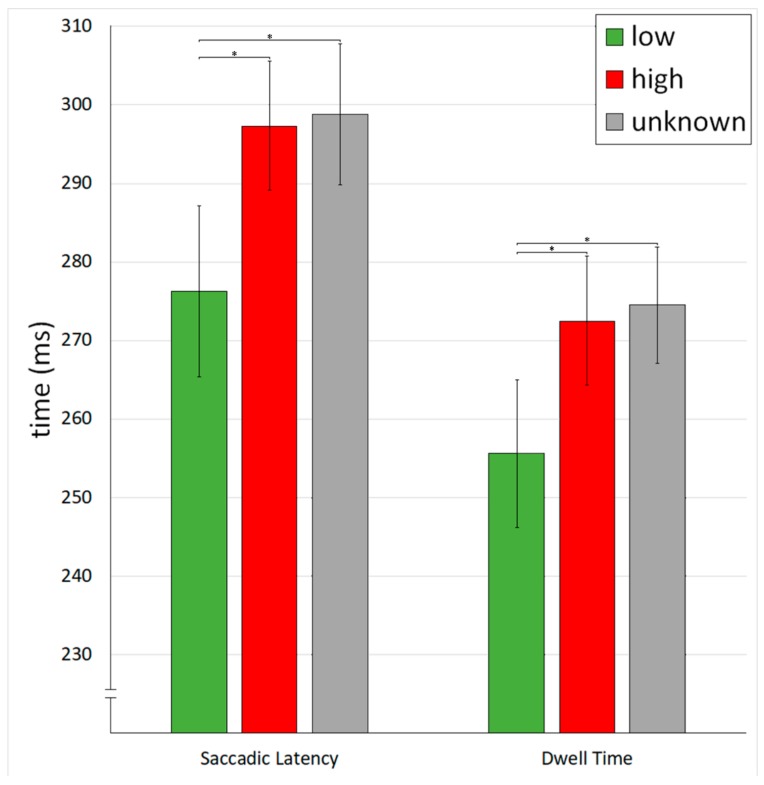
Mean saccadic latency and dwell time for trials in which food appeared in the current gaze location for three CNI conditions: Low (green CNI/low sugar), high (red CNI/high sugar), and unknown (gray CNI/unknown sugar). Whiskers indicate standard errors. Asterisks indicate Holm-adjusted *p* < 0.05.

Saccadic Latency*:* For gaze relocation (same position), the repeated measures ANOVA revealed a significant main effect of CNI on saccadic latency (*F*(1.71,85.55) = 4.98, *p* = 0.012, *η*2*p* = 0.091). The saccadic latency was significantly lower for food with a low sugar content compared to food with a high sugar content (*t*(50) = 2.35, *p* = 0.045, *d* = 0.33) and unknown sugar content (*t*(50) = 2.63, *p* = 0.034, *d* = 0.37). The saccadic latency did not differ between unknown and high sugar content (*t*(50) = 0.24, *p* = 0.82, *d* = 0.03; see Figure 2).

Dwell Time: The ANOVA revealed a significant main effect of CNI on the dwell time spent on the food images (*F*(1.61,80.48) = 5.61, *p* = 0.009, *η*2*p* = 0.10). The dwell time was shorter for food with a low sugar content compared to food with a high sugar content (*t*(50) = 2.37, *p* = 0.043, *d* = 0.33) and unknown sugar content (*t*(50) = 2.83, *p* = 0.020, *d* = 0.40). Dwell time did not differ between unknown and high sugar content (*t*(50) = 0.44, *p* = 0.66, *d* = 0.06; see Figure 2 and Table 2).

For gaze avoidance (peripheral position), there was no significant effect of CNI on dwell time (*F*(2,100) = 1.70, *p* = 0.19, *η*2*p* = 0.03). The dwell time did not differ significantly between low sugar content (*M* = 71.5, SD = 61.1), high sugar content (*M* = 58.4, SD = 52.1), and unknown sugar content (*M* = 65.0, SD = 49.1).

#### 3.1.4. Exploratory Analysis

To analyze if the general preference for sweet foods was correlated with CNI, we calculated Pearson correlations between liking of sweet foods and (1) the difference in saccadic latency between high and low sugar content (high sugar saccadic latency minus low sugar saccadic latency) and with (2) the difference in dwell time (high sugar dwell time minus low sugar dwell time). On average, the reported liking was *M* = 2.86 (SD = 1.02). We found positive correlations between liking and difference in saccadic latency (*r* = 0.298, *p* = 0.034) and dwell time (*r* = 0.369, *p* = 0.008).

The difference in saccadic latency was not correlated with the I-8 score (*r* = 0.022, *p* = 0.879), the EDE-Q score (*r* = 0.217, *p* = 0.126), the BMI (*r* = 0.01, *p* = 0.95), hunger (*r* = 0.169, *p* = 0.236), or appetite (*r* = 0.055, *p* = 0.700). Also, the difference in dwell time was not correlated with the I-8 score (*r* = −0.081, *p* = 0.570), the EDE-Q (*r* = 0.247, *p* = 0.081), the BMI (*r* = 0.08, *p* = 0.58), hunger (*r* = 0.103, *p* = 0.474), or appetite (*r* = −0.003, *p* = 0.982).

### 3.2. Results Experiment 2

We calculated two ANOVAs to test the effects of color (red, green, gray circles) on estimated sweetness and valence of the food stimuli. We found no significant color effect for sweetness *F*(2,196) = 0.22, *p* = 0.81, *η*2*p* = 0.002 (Table 2). The effect for valence was significant, *F*(2,196) = 3.16, *p* = 0.045, *η2p* = 0.031 (Table 2). The post-hoc pairwise comparisons were not significant (all *p* > 0.08). Food items preceded by a gray circle received marginally higher valence ratings compared to red circles.

## 4. Discussion

The shopping of food, including high-calorie sweet snack foods, is often impulsive. In order to influence this spontaneous shopping behavior, simple interventions are needed that are able to interrupt this process. The current eye-tracking study investigated the influence of provided information about a product’s sugar content on visual food cue reactivity. It was tested whether a red circle that indicated a high sugar content of a product would be able to help the participants to direct their gaze away from the displayed food item. The results showed that the intervention had the opposite of the intended effect. The dwell time and the saccadic latency were lower for food items preceded by a green circle compared to a red and gray circle. Obviously, it was easier for the participants to ignore food cues if low sugar content was assumed relative to high or unknown sugar content. Thus, the participants showed a paradox reaction.

Similar paradox effects have been reported in studies that attempted to influence knowledge and beliefs about food [39,40]. A study by Berry et al. [39] examined how calorie information on menus in chain restaurants affected the food choice. The results indicated that calorie labeling even increased the calories ordered if the consumers were taste-oriented rather than health-oriented. Similarly, Provencher et al. [40] found that participants ate 30% more of the same cookies when labeled as healthy.

Whereas the current study contributes to the existing evidence that nutrition facts may be ineffective [39,40,41,42], other findings have indicated that nutrition fact information provided via food labels is a useful tool to target food cue reactivity and food choices [28,43,44]. Further research is needed to evaluate in which cases unintended effects of CNI on food cue reactivity might occur. Our exploratory analysis indicated that it was more difficult for participants with a high compared to a low preference for sweet foods to avoid ‘high sugar’ foods. Thus, individual preferences might overrule CNI [41].

Additionally, previous research has indicated that the color-coding itself may elicit unintended effects on FCR. Cross-modal associations between the color red and sweet taste have been reported in many studies. Cross-modal associations were observed primarily for fluids [30] and not for solid foods [45]. The present study (with exclusively solid foods) found no evidence for priming effects of red on estimated sweetness and pleasantness of the depicted food products. The food items even received marginally higher valence ratings after the presentation of a gray circle compared to a red circle. Thus, it is unlikely that the results of Experiment 1 were caused by cross-modal associations between priming color and visual food perception.

We need to mention the following limitations of the present study. In Experiment 1, we only studied female participants. The majority of the women were university students. Therefore, our findings cannot be generalized to other samples. However, it is important to note that we used an innovative gaze performance task to evaluate visual food cue reactivity without the possible effects of self-monitored gaze direction or social desirability (as opposed to free exploration paradigms and self-reports). The task was very easy and therefore should have been accomplished by this group of highly educated women. Nevertheless, to determine if the basic findings of the present study can be applied to other participants and circumstances (e.g., male and/or less-educated participants), a replication study is highly recommended. Experiment 2 was not conducted in the lab, but at home. Thus, we were not able to control unintended distractions during participation.

## Figures and Tables

**Table 1:** Sample characteristics and rating data.

Measure	Mean (SD)
Age (years)	22.04 (2.99)
BMI	22.47 (3.85)
Hunger level (0–6)	1.47 (1.52)
General appetite (0–6)	1.86 (1.55)
Sweet food preference (1–4)	2.86 (1.02)
Specific appetite (0–6)	
low sugar	2.06 (1.34)
high sugar	2.11 (1.26)
unknown sugar	2.22 (1.38)
General liking (0–6)	
low sugar	3.51 (1.48)
high sugar	3.64 (1.29)
unknown sugar	3.77 (1.27)

**Table 2:** Summary results of Experiments 1 and 2.

Measure	ANOVA	Green (SD)	Red (SD)	Gray (SD)
**Experiment 1**:				
Saccadic latency	*F*(1.71,85.55) = 4.98, *p* = 0.012*, η*2*p* = 0.091	276.3 ms (77.4 ms)	297.4 ms (58.6 ms)	398.8 ms (64.4 ms)
Dwell time current *	*F*(1.61,80.48) = 5.61, *p* = 0.009, *η*2*p* = 0.10	255.6 ms (67.3 ms)	272.5 ms (58.8 ms)	274.5 ms (52.9 ms)
Dwell time peripheral *	*F*(2,100) = 1.70, *p* = 0.19, *η*2*p* = 0.03	71.5 ms (61.1 ms)	58.4 ms (52.1 ms)	65.0 ms (49.1 ms)
**Experiment 2**:				
Sweetness	*F*(2,196) = 0.22, *p* = 81, *η*2*p* = 0.002	74.4% (10.9%)	74.0% (11.1%)	74.0% (10.8%)
Valence *	*F*(2,196) = 3.16, *p* = 045, *η*2*p* = 0.031	44.7% (21.1%)	43.1% (19.7%)	49.6% (22.2%)

In Experiment 1, green indicated low sugar, red indicated high sugar, and gray indicated no specific sugar content. For gaze data (Experiment 1), mean durations in milliseconds are given. Sweetness and valence were rated from 0% to 100%. Asterisks indicate significant main effects.
